# Higher visceral fat area increases the risk of vitamin D insufficiency and deficiency in Chinese adults

**DOI:** 10.1186/s12986-015-0046-x

**Published:** 2015-11-25

**Authors:** Meilin Zhang, Ping Li, Yufeng Zhu, Hong Chang, Xuan Wang, Weiqiao Liu, Yuwen Zhang, Guowei Huang

**Affiliations:** Department of Nutrition and Food Science, School of Public Health, Tianjin Medical University, Tianjin, 300070 China; Department of Rehabilitation and Sports Medicine, Tianjin Medical University, Tianjin, 300070 China; Health Education and Guidance Center of Heping District, Tianjin, 300040 China

**Keywords:** Visceral fat area, 25-hydroxyvitamin D_3_, Vitamin D deficiency, Visceral fat obesity

## Abstract

**Background:**

Visceral fat area (VFA), a novel sex-specific index for visceral fat obesity (VFO) might play a major role in the development of vitamin D deficiency. However, the association between VFA and vitamin D insufficiency and deficiency in Chinese population is less clear. The aim of this study was to explore the population-level association between VFA and vitamin D insufficiency and deficiency among Chinese men and women.

**Methods:**

This cross-sectional study involved 1105 adults aged 20–70 years living in Tianjin who were randomly selected and medically examined. All subjects underwent the bioelectrical impedance analysis (BIA) method to estimate the VFA. Serum 25-hydroxyvitamin D_3_ (25(OH) D_3_) level was assayed by the high-performance liquid chromatography (HPLC) method and defined insufficiency and deficiency following recommended cutoffs. The association between VFA and vitamin D insufficiency and deficiency was estimated using binary regression analysis.

**Results:**

The total prevalence of vitamin D insufficiency (25(OH) D_3_: 20–29 μg/L) and deficiency (25(OH) D_3_ < 20 μg/L) were 26.60 % and 24.89 %, respectively. Significant negative association was observed for VFA with serum 25(OH) D_3_ levels in men and pre-menopausal women (*P* < 0.05), not in post-menopausal women (*P* > 0.05). Moreover, increased VFA was observed to be associated with higher vitamin D insufficiency or deficiency risk with a positive dose–response trend (*P* for trend < 0.001). As compared to individuals with the lowest VFA, those who had the highest VFA were at 4.9-fold risk of vitamin D insufficiency and deficiency [95 % confidence interval (95 % CI): 1.792–13.365] in men and 1.8-fold risk of vitamin D insufficiency and deficiency (95 % CI: 1.051–3.210) in pre-menopausal women, but not in post-menopausal women [odds ratio (OR) (95 % CI): 2.326(0.903–5.991)].

**Conclusions:**

These results suggest that higher VFA increases the risk of vitamin D insufficiency and deficiency in men and pre-menopausal women, but not in post-menopausal women. VFA is a better and convenience surrogate marker for visceral adipose measurement and could be used in identifying the risk of vitamin D insufficiency and deficiency in routine health examination.

## Background

Vitamin D deficiency is now being recognized as a major global public health problem [1–3]. Approximately 1 billion people worldwide have vitamin D deficiency [4]. In China, vitamin D deficiency is common in the middle-aged and elderly population. The prevalence of vitamin D deficiency was 69.2 % in Beijing and Shanghai [5], and the prevalence of vitamin D deficiency was 75.2 % in the northwestern inland of China [6].

Traditional roles of vitamin D lie within the musculoskeletal system, maintaining calcium homoeostasis and bone metabolism. In recent years, new functional roles of vitamin D have emerged linking the fat-soluble vitamin to various non-communicable diseases. The consequences of vitamin D deficiency include poor bone development and health as well as increase risk of many chronic diseases including insulin resistance, diabetes and cardiovascular disease [7], which is also commonly linked with obesity and visceral fat obesity (VFO).

Anthropometric indices such as body mass index (BMI) have been used widely in the previous studies examining the relationship between obesity and vitamin D [8, 9]. In particular, VFO compared with overall obesity might play a major role in the development of vitamin D deficiency. Therefore, estimating visceral fat accumulation is important to identify individuals at high risk for vitamin D deficiency. Their inability to distinguish visceral fat precludes their ability to accurately assess abdominal fat accumulation. Because fat accumulation in different areas represents different risk for metabolic disorders and visceral adipose tissue may be unique [10], we sought to evaluate visceral fat. Conventionally, the simplest and most widely used method of assessing visceral fat accumulation is measuring the waist circumference (WC) [11]. Although WC is recognized as an independent and powerful risk factor for vitamin D deficiency, there might be substantial variations in the visceral fat amount among persons with a similar WC because WC itself performs poorly in discriminating between visceral and subcutaneous fat. Therefore, it is possible that subjects with a normal WC may be more prone to vitamin D deficiency if they have centrally located body fat. Many sophisticated methods such as dual-energy x-ray absorptiometry (DEXA), computed tomography (CT) and magnetic resonance imaging (MRI) have been developed to provide more precise estimates of the location and amount of adipose tissue in various body regions [12]. However, DEXA, CT and MRI are impractical for screening general population, since they require expensive and specialized equipment, and exposure to radiation. Studies have compared the general usage of bioelectrical impedance analysis (BIA) with one of these more established methods (CT, MRI or DEXA). BIA has been shown to accurately predict DXEA-derived percentage body fat (PBF) in the Chinese population (r = 0.89, *P* < 0.05) [13]. VFA measured by BIA correlated significantly with that acquired by CT (r = 0.992 for VFA, using data from the Biospace of 332 patients) [14]. Although BIA is not considered a “gold standard”, BIA has been considered a valid alternative for measuring body fat in large studies or clinical practice [15], which has also been validated against reference methods [13, 14].

Visceral fat area (VFA), a novel sex-specific index for VFO, has been proposed recently. One study has examined the association of 25-hydroxyvitamin D_3_ (25(OH) D_3_) level and VFO in Chinese men and discovered serum 25(OH)D_3_ levels were inversely associated with VFA [16]. To our knowledge, serum 25(OH) D_3_ levels and the distribution of abdominal fat are known to be distinctive between men and women [17–19], as well as between pre-memopausal and post-memopausal women. However, the significance of association between VFA and vitamin D insufficiency and deficiency in the Chinese population is less clear. In the present study, a simple method for the measurement of visceral fat accumulation using BIA [20] was performed to explore the population-level association between VFA and vitamin D insufficiency and deficiency in Chinese men and women.

## Methods

### Subjects

This overall population includes 1130 subjects aged 20–70 years coming to the Health Examination Center of Heping District in Tianjin, China for routine health checkup during November to December in 2013, were enrolled. 1105 subjects were included for analysis according to the following inclusion and exclusion criteria. Inclusion criteria were as follows: 1) aged 20–70 years, 2) written informed consent, 3) willingness to take part in the examination and willingness to provide blood samples. Exclusion criteria were as follows: 1) individuals whose medical data were incomplete (*n* = 10), 2) individuals who took diuretics (*n* = 3), 3) individuals who undergone hormone replacement therapy (*n* = 2), 4) individuals with chronic kidney disease (*n* = 3), 5) individuals with presence of tumor, infectious diseases or psychiatric disease (*n* = 3), 6) individuals with severe disability or occurrence of bone fracture within the past six months (*n* = 2); 7) individuals who have had stents or with history of cardiovascular disease (*n* = 2).

The study was conducted in accordance with the Declaration of Helsinki and approved by the Ethics Committee of Tianjin Medical University. All study participants provided written informed consent prior to enrollment.

### Body fat measurements

Body fat measurements, consisting of BMI, WC, waist-to-hip ratio (WHR), fat mass (FM), free fat mass (FFM), PBF and VFA were estimated by the BIA method (Inbody 720 Body Composition Analyzer, Korean).

### Anthropometric and biochemical assessments

Systolic blood pressure (SBP) and diastolic blood pressure (DBP) were measured by trained nurses with a mercury sphygmomanometer on the right arm of the subjects in a comfortable siting position after 5 -min rest. After 10-h overnight fasting, blood samples were collected from all subjects to measure serum 25(OH) D_3_ levels as well as other biochemical parameters. Fasting plasma glucose (FPG) was measured by the glucose oxidase method. Lipid profiles, including total cholesterol (TC) and triglycerides (TG) were determined using the standard enzymatic methods, while low-density lipoprotein cholesterol (LDL-c) and high-density lipoprotein cholesterol (HDL-c) were determined by the direct assay method. All of the above measurements were carried out on a Hitachi 7600–120 auto-analyzer (Tokyo, Japan). Total serum 25(OH) D_3_ level was assayed by the high-performance liquid chromatography (HPLC) method.

### Definition of terms

Obesity was defined as a BMI value exceeding 28 kg/m^2^ [21]. VFO was defined as WC ≥ 90 cm for men or ≥80 cm for women. Or VFO was defined as a VFA exceeding 130 cm^2^ in men and 100 cm^2^ in women [22]. Vitamin D status was defined as follows: A serum level of 25(OH) D_3_ < 20 μg/L is considered to be vitamin D deficiency, whereas a level 20–29 μg/L is insufficient, and to maximize vitamin D’s effect for health, 25(OH) D_3_ should be ≥30 μg/L [23].

### Lifestyle factors

Smoking behavior, drinking behavior, current daily time spent outdoors, physical activity, menopausal status and vitamin D supplement intake were collected using self-administered questionnaires. The current time spent outdoors (h/d) was used as an indicator for sunshine time. A woman was considered post-menopausal if she reported menses had ceased for 1 year or more, and age at menopause was self-reported as previously reported.

### Statistical analysis

According to the sample size, a normal distribution was tested by the Kolmogorov-Smirnov test with the Lilliefors correction for men and women. Normally distributed variables were presented as the mean ± standard deviation, whereas skewed variables were presented as the median (interquartile range). Because of significant sex difference in the amount of total body fat, fat distribution and lifestyle factors, we performed sex-stratified analyses. For continuous variables, the independent sample *t*-test and the Wilcoxon rank-sum test were used for between-group comparisons of normally distributed and skewed data, respectively. Comparative analyses of categorical variables were carried out using a Chi-square test. Each participant was categorized into one of four subgroups depending on their total VFA. To compare variables between these subgroups, one-way analysis of variance (ANOVA) followed by Scheffé post hoc test or Mann–Whitney *U* test were used where appropriate. Partial correlation analyses between serum 25(OH) D_3_ and other variables were adjusted for age. Binary logistic regression was performed to examine the association between body fatness indices and vitamin D insufficiency and deficiency. Data were analyzed using SPSS (Chicago, IL) version 13.0 statistical software. *P* value less than 0.05 was considered to indicate a statistically significant difference.

## Results

### Descriptive characteristics of the study population

The characteristics of the study population were shown in Table 1. Overall, the prevalence of visceral fat obesity was higher than obesity. It was showed that the men had a higher age, WHR, BMI, WC, FM, VFA, FFM, SBP, DBP, sunshine time, higher levels of TG, LDL-c, FPG and lower levels of PBF, 25(OH) D_3_ and HDL-c. But there was no difference in TC and vitamin D supplementation intake between the men and women. The prevalence of obesity, visceral fat obesity and vitamin D deficiency in men was higher than in the women, whereas the prevalence of vitamin D insufficiency in men was lower than in the women.

**Table 1:** Descriptive characteristics of the study population

Characteristics	Total	Men	Women	*P*-value^a^
*n*	1105	257	848	
Age, y	43.82 ± 12.15	45.44 ± 12.18	43.33 ± 12.11	0.015
Body mass index, kg/m^2^	23.67 ± 3.37	26.18 ± 3.39	22.90 ± 2.97	<0.001
Waist circumference, cm	82.79 ± 9.08	90.42 ± 8.57	80.48 ± 7.90	<0.001
Waist-to- hip ratio	0.87 ± 0.05	0.91 ± 0.04	0.84 ± 0.04	<0.001
Fat mass, kg	20.13 ± 6.06	21.45 ± 6.91	19.73 ± 5.72	<0.001
Free fat mass, kg	44.66 ± 8.96	58.41 ± 6.56	40.50 ± 4.11	<0.001
Percentage body fat, %	30.89 ± 6.06	26.35 ± 5.57	32.27 ± 5.51	<0.001
Visceral fat area, cm^2^	87.30 ± 35.44	116.13 ± 34.87	78.56 ± 30.68	<0.001
Systolic blood pressure, mmHg	113.79 ± 15.72	120.54 ± 12.72	111.07 ± 16.01	<0.001
Diastolic blood pressure, mmHg	75.52 ± 10.06	80.30 ± 9.34	73.59 ± 9.70	<0.001
Total cholesterol, mmol/L	4.98 ± 0.98	5.03 ± 0.95	4.97 ± 1.00	^b^NS
Triglycerides, mmol/L	1.03(0.73–1.51)	1.41(0.99–2.00)	0.95(0.69–1.36)	<0.001
High-density lipoprotein cholesterol, mmol/L	1.33 ± 0.28	1.16 ± 0.21	1.38 ± 0.28	<0.001
Low-density lipoprotein cholesterol, mmol/L	2.96 ± 0.80	3.10 ± 0.76	2.92 ± 0.82	0.001
Fasting plasma glucose, mmol/L	5.73 ± 1.03	5.96 ± 1.28	5.66 ± 0.92	<0.001
25(OH)D_3_, μg/L	36.46 ± 24.70	33.26 ± 19.80	37.44 ± 25.94	0.018
Smoking, % yes	11.86	42.80	2.48	<0.001
Drinking, % yes	38.82	70.04	29.36	<0.001
Doing exercise, % yes	46.06	64.98	40.33	<0.001
Sunshine time, h/day	0.5(0.0,1.0)	1.0(0.0,2.0)	0.5(0.0,1.0)	<0.001
Vitamin D supplement users, % yes	2.26	2.33	2.24	^b^NS
Obesity, %	13.76	29.18	9.08	<0.001
Visceral fat obesity, %				
Men: WC ≥90 cm; women: WC ≥ 80 cm	44.71	50.58	42.92	0.031
Men: VFA ≥130 cm^2^; women: VFA ≥ 100 cm^2^	23.53	35.40	19.92	<0.001
25(OH)D_3_ status^*^				
Vitamin D insufficiency, %	26.60	18.68	29.00	0.001
Vitamin D deficiency, %	24.89	33.46	22.29	<0.001

Values are percentages for categorical variables, means ± SDs for continuous variables with a normal distribution, or medians (95 % ranges) for continuous variables with a skewed distribution

*Abbreviations*: *WC*, waist circumference; *VFA*, visceral fat area; 25(OH)D_3_, 25-hydroxyvitamin D

^a^Comparison among men and women

^b^NS = no significant at *p* value > .05

^*^25(OH)D_3_ status was defined as follows: A serum level of 25(OH) D_3_ < 20 μg/L is considered to be vitamin D deficiency, whereas a level 20–29 μg/L is insufficient

### Characteristics of the study population according to the quartiles of VFA

The study subjects were also divided into four groups according to the quartiles of VFA in men and women. The analysis revealed a increasing trend in VFA that accompanied increases in BMI, WC, WHR, FM, FFM, PBF and TG in both genders, decreases in 25(OH)D_3_ in men and pre-menopausal women (Table 2, Table 3). Intergroup comparisons revealed that the Q2, Q3 and Q4 groups had significantly lower 25(OH) D_3_ than the Q1 group in men (Table 2), whereas the Q4 groups had significantly lower 25(OH) D_3_ than the Q2 group and Q1 group in pre-menopausal women (Table 3).

**Table 2:** Characteristics of the study population according to quartiles of visceral fat area levels in men

	Quartiles of visceral fat area (cm^2^)				*P*-value^a^
	Q1 (<89.95)	Q2 (89.95–115.72)	Q3 (115.72–140.46)	Q4 (≥140.46)	
*n*	64	65	64	64	
Age, y	42.36 ± 12.69	45.29 ± 12.03	46.92 ± 12.06	47.17 ± 11.58	^b^NS
Body mass index, kg/m^2^	22.87 ± 2.23	25.59 ± 2.21^#^	26.80 ± 2.51^#^^&^	29.45 ± 2.77^#^^&^^$^	0.000
Waist circumference, cm	81.28 ± 5.58	88.36 ± 4.93^#^	92.48 ± 5.50^#^^&^	99.57 ± 5.83^#^^&^^$^	0.000
Waist-to-hip ratio	0.86 ± 0.03	0.90 ± 0.02^#^	0.92 ± 0.02^#^^&^	0.95 ± 0.02^#^^&^^$^	0.000
Fat mass, kg	14.08 ± 3.52	19.90 ± 3.67^#^	23.35 ± 4.97^#^^&^	28.51 ± 5.64^#^^&^^$^	0.000
Free fat mass, kg	55.38 ± 6.43	57.40 ± 6.06^#^	58.17 ± 5.60^#^^&^	62.75 ± 5.93^#^^&^^$^	0.000
Percentage body fat, %	20.14 ± 3.63	25.68 ± 3.39^#^	28.50 ± 4.28^#^^&^	31.08 ± 4.02^#^^&^^$^	0.000
Systolic blood pressure, mmHg	118.89 ± 11.35	121.78 ± 12.12	118.41 ± 11.35	122.93 ± 14.70	^b^NS
Diastolic blood pressure, mmHg	78.19 ± 7.57	80.22 ± 10.05	80.57 ± 8.16	81.95 ± 10.95	^b^NS
Total cholesterol, mmol/L	4.71 ± 0.78	4.93 ± 1.08	5.17 ± 0.90^#^	5.31 ± 0.90^#^^&^	0.001
Triglycerides, mmol/L	0.99(0.72–1.46)	1.38(0.94–2.10)^#^	1.53(1.22–2.11)^#^	1.69(1.26–2.18)^#^	0.036
High-density lipoprotein cholesterol, mmol/L	1.22 ± 0.23	1.14 ± 0.21	1.13 ± 0.21	1.15 ± 0.18	^b^NS
Low-density lipoprotein cholesterol, mmol/L	2.85 ± 0.66	2.91 ± 0.68	3.24 ± 0.76^#^^&^	3.44 ± 0.79^#^^&^	0.000
Fasting plasma glucose, mmol/L	5.63 ± 0.95	6.10 ± 1.36	5.99 ± 1.13	6.13 ± 1.58	^b^NS
25(OH)D_3_, μg/L	42.30 ± 19.59	34.90 ± 20.93^#^	30.80 ± 20.15^#^	25.01 ± 14.05^#^^&^	0.000
Smoking, % yes	28.13	41.54^#^	43.75^#^	57.81^#^	0.009
Drinking, % yes	57.81	73.85	71.88	76.56	^b^NS
Doing exercise, % yes	51.56	67.69	67.19	73.44	^b^NS
Sunshine time, h/day	1.00(0.50–1.00)	1.00(0.00–3.00)	0.50(0.00–1.50)	1.00(0.00–2.00)	^b^NS
Vitamin D supplement users, % yes	1.56	0.00	6.25	1.56	^b^NS

Values are percentages for categorical variables, means ± SDs for continuous variables with a normal distribution, or medians (95 % ranges) for continuous variables with a skewed distribution

Abbreviations: 25(OH) D_3_, 25–hydroxyvitamin D_3_

^a^Comparison among four groups

^b^NS = no significant at *p* value > .05

^#^Compared with Q1, *P* < 0.05

^&^Compared with Q2, *P* < 0.05

^$^Compared with Q3, *P* < 0.05

**Table 3:** Characteristics of the study population according to quartiles of visceral fat area levels in women

	Pre-menopausal women				
	Quartiles of visceral fat area (cm^2^)				*P*-value^a^
	Q1 (<53.15)	Q2 (53.15–66.18)	Q3 (66.18–84.47)	Q4 (≥84.47)	
*n*	153	144	163	153	
Age, y	33.09 ± 6.30	36.54 ± 7.54^#^	38.35 ± 6.91^#^^&^	41.27 ± 7.41^#^^&^^$^	0.000
Body mass index, kg/m^2^	20.51 ± 1.33	21.54 ± 1.46^#^	22.44 ± 1.77^#^^&^	25.48 ± 3.46^#^^&^^$^	0.000
Waist circumference, cm	73.06 ± 3.74	76.67 ± 3.48^#^	79.62 ± 3.92^#^^&^	88.27 ± 7.90^#^^&^^$^	0.000
Waist-to-hip ratio	0.81 ± 0.02	0.84 ± 0.02^#^	0.86 ± 0.02^#^^&^	0.90 ± 0.04^#^^&^^$^	0.000
Fat mass, kg	14.39 ± 2.71	17.02 ± 2.90^#^	18.99 ± 3.08^#^^&^	24.93 ± 5.80^#^^&^^$^	0.000
Free fat mass, kg	39.65 ± 3.46	39.84 ± 3.69	40.16 ± 3.42	43.23 ± 4.85^#^^&^^$^	0.000
Percentage body fat, %	26.53 ± 3.69	29.85 ± 3.74^#^	32.01 ± 3.58^#^^&^	36.26 ± 4.46^#^^&^^$^	0.000
Systolic blood pressure, mmHg	102.64 ± 12.01	102.31 ± 10.81	106.97 ± 112.46^#^	110.85 ± 15.45^#^^&^	0.000
Diastolic blood pressure, mmHg	69.03 ± 8.46	69.18 ± 7.37	72.13 ± 8.73	74.38 ± 10.18^#^^&^	0.001
Total cholesterol, mmol/L	4.42 ± 0.72	4.63 ± 0.77^#^	4.73 ± 0.87^#^	4.90 ± 0.87^#^^&^^$^	0.000
Triglycerides, mmol/L	0.66(0.58–0.86)	0.79(0.64–1.07)^#^	0.94(0.69–1.25)^#^^&^	1.16(0.80–1.55)^#^^&^^$^	0.000
High-density lipoprotein cholesterol, mmol/L	1.46 ± 0.29	1.41 ± 0.30	1.32 ± 0.24^#^^&^	1.26 ± 0.25^#^^&^	0.000
Low-density lipoprotein cholesterol, mmol/L	2.41 ± 0.62	2.64 ± 0.63^#^	2.81 ± 0.76^#^^&^	3.02 ± 0.75^#^^&^^$^	0.000
Fasting plasma glucose, mmol/L	5.29 ± 0.47	5.37 ± 0.45	5.55 ± 0.78^#^^&^	5.77 ± 0.90^#^^&^^$^	0.000
25(OH)D_3_, μg/L	43.25 ± 28.90	42.30 ± 28.02	38.39 ± 24.11	35.80 ± 27.38^#^^&^	0.002
Smoking, % yes	0.65	0.69	0.61	0.65	^b^NS
Drinking, % yes	22.22	25.00	24.54	29.41	^b^NS
Doing exercise, % yes	44.44	48.61	43.56	47.06	^b^NS
Sunshine time, h/day	1.00(0.50–0.10)	1.00(0.00–1.00)	1.00(0.50–0.10)	1.00(0.50–0.10)	^b^NS
Vitamin D supplement users, % yes	1.96	0.69	1.23	0.65	^b^NS
	Post-menopausal women				
	Quartiles of visceral fat area (cm^2^)				*P*-value^a^
	Q1(<71.55)	Q2(71.55–91.97)	Q3(91.97–115.73)	Q4(≥115.73)	
*n*	59	59	59	58	
Age, y	58.17 ± 7.74	59.25 ± 5.49	58.95 ± 5.60	59.38 ± 5.81	^b^NS
Body mass index, kg/m^2^	21.43 ± 1.60	22.62 ± 1.22^#^	24.18 ± 2.00^#^^&^	27.64 ± 2.51^#^^&^^$^	0.000
Waist circumference, cm	75.57 ± 4.19	79.47 ± 2.73^#^	84.24 ± 4.41^#^^&^	93.64 ± 6.10^#^^&^^$^	0.000
Waist-to-hip ratio	0.82 ± 0.02	0.86 ± 0.01^#^	0.89 ± 0.01^#^^&^	0.93 ± 0.03^#^^&^^$^	0.000
Fat mass, kg	16.80 ± 2.85	19.42 ± 2.35^#^	22.66 ± 3.51^#^^&^	29.18 ± 4.87^#^^&^^$^	0.000
Free fat mass, kg	38.72 ± 3.89	38.79 ± 2.88	39.66 ± 3.24	42.51 ± 4.45^#^^&^^$^	0.000
Percentage body fat, %	30.18 ± 3.77	33.34 ± 3.03^#^	36.25 ± 3.45^#^^&^	40.55 ± 3.97^#^^&^^$^	0.000
Systolic blood pressure, mmHg	115.14 ± 14.90	120.78 ± 18.67	122.36 ± 13.65	124.88 ± 15.37	^b^NS
Diastolic blood pressure, mmHg	75.97 ± 9.47	76.72 ± 8.67	77.22 ± 8.23	81.63 ± 10.04^#^^&^^$^	0.029
Total cholesterol, mmol/L	5.64 ± 0.93	5.84 ± 0.10	5.88 ± 1.17	5.58 ± 0.82	^b^NS
Triglycerides, mmol/L	1.10(0.67–1.40)	1.21 (0.93–1.50)^#^	1.40(1.07–1.94)^#^^&^	158(1.16–1.89)^#^^&^	0.000
High-density lipoprotein cholesterol, mmol/L	1.55 ± 0.30	1.42 ± 0.23^#^	1.33 ± 0.25^#^	1.35 ± 0.26^#^	0.000
Low-density lipoprotein cholesterol, mmol/L	3.31 ± 0.80	3.57 ± 0.73	3.54 ± 0.85	3.33 ± 0.81	^b^NS
Fasting plasma glucose, mmol/L	5.75 ± 1.34	6.06 ± 1.13	6.35 ± 1.51	6.10 ± 0.84	^b^NS
25(OH)D_3_, μg/L	33.04 ± 22.62	33.63 ± 20.89	29.77 ± 20.90	27.78 ± 19.64	^b^NS
Smoking, % yes	3.39	6.78	8.47	10.34	^b^NS
Drinking, % yes	33.90	38.98	40.68	46.55	^b^NS
Doing exercise, % yes	32.20	30.51	22.03	18.97	^b^NS
Sunshine time, h/day	1.00(0.50–0.10)	1.00(0.00–1.00)	1.00(0.50–0.10)	1.00(0.50–0.10)	^b^NS
Vitamin D supplement users, % yes	3.39	5.08	6.78	5.17	^b^NS

Values are percentages for categorical variables, means ± SDs for continuous variables with a normal distribution, or medians (95 % ranges) for continuous variables with a skewed distribution

Abbreviations: 25(OH) D_3_, 25-hydroxyvitamin D_3_

^a^Comparison among four groups

^b^NS = no significant at *p* value > .05

^#^Compared with Q1, *P* < 0.05

^&^Compared with Q2, *P* < 0.05

^$^Compared with Q3, *P* < 0.05

### Prevalence of vitamin D insufficiency and vitamin D deficiency according to the quartiles of VFA

**Fig. 1:**
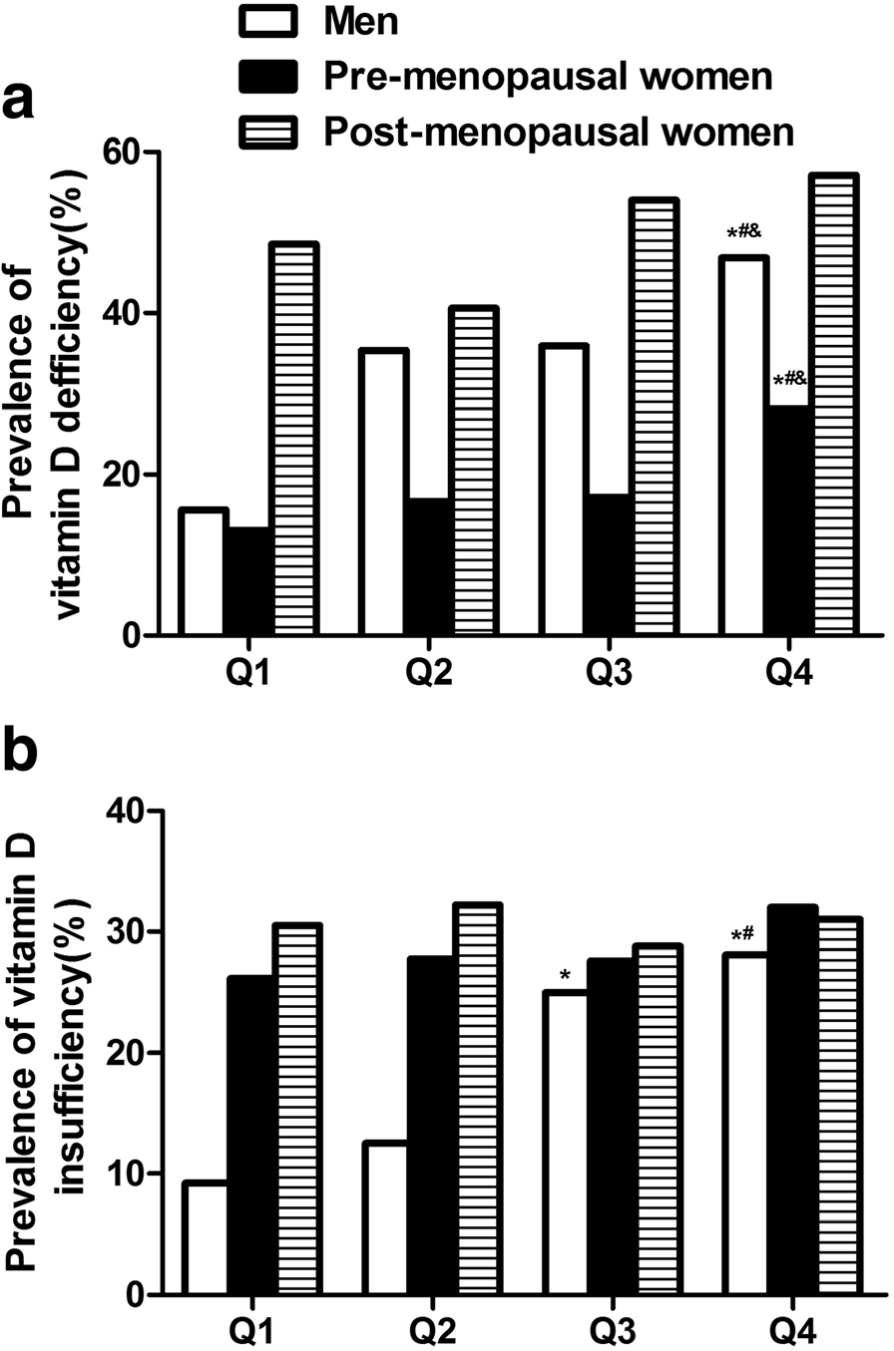
Prevalence of vitamin D deficiency (25(OH) D_3_) <20 μg/L) (**a**) or vitamin D insufficiency (25(OH) D_3_: 20–29 μg/L) (**b**) according to the quartiles of visceral fat area in men and women. Men (Q1: <89.95 cm^2^, Q2: 89.95–115.72 cm^2^, Q3: 115.72–140.46 cm^2^, Q4: ≥140.46 cm^2^); Pre-menopausal women (Q1: <53.15 cm^2^, Q2: 53.15–66.18 cm^2^, Q3: 66.18–84.47 cm^2^, Q4: ≥ 84.47 cm^2^); Post-menopausal women (Q1: <71.55 cm^2^, Q2: 71.55–91.97 cm^2^, Q3: 91.97–115.73 cm^2^, Q4: ≥115.73 cm^2^). ^*^Compared with Q1, *P* <0.05; ^#^Compared with Q2, *P* <0.05; ^&^Compared with Q3, *P* <0.05

Figure 1a showed that the prevalence of vitamin D deficiency increased in men and in pre-menopausal women according to the quartiles of VFA (*P* <0.05), but not in post-menopausal women (*P* >0.05). The prevalence of vitamin D deficiency in the fourth quartile of VFA in men (46.88 %) and in pre-menopausal women (28.10 %) were significantly higher than that in the first quartile (15.63 % in men and 13.07 % in pre-menopausal women), second quartile (35.38 % in men and 16.67 % in pre-menopausal women) and third quartile (35.94 % in men and 17.18 % in pre-menopausal women) (*P* <0.05) (Fig. 1a). Increased VFA was observed to be associated with higher vitamin D deficiency risk with a positive dose–response trend in men and pre-menopausal women (*P* for trend < 0.001), not in post-menopausal women. The prevalence of vitamin D insufficiency in the fourth quartile of VFA in men (28.13 %) was significantly higher than that in the first quartile (9.23 %) and second quartile (12.50 %) (Fig. 1b). However, the prevalence of vitamin D insufficiency did not increase with the quartiles of VFA in pre-menopausal women and post-menopausal women (*P* >0.05) (Fig. 1b).

### Relationship between VFA and vitamin D insufficiency and deficiency

**Fig. 2:**
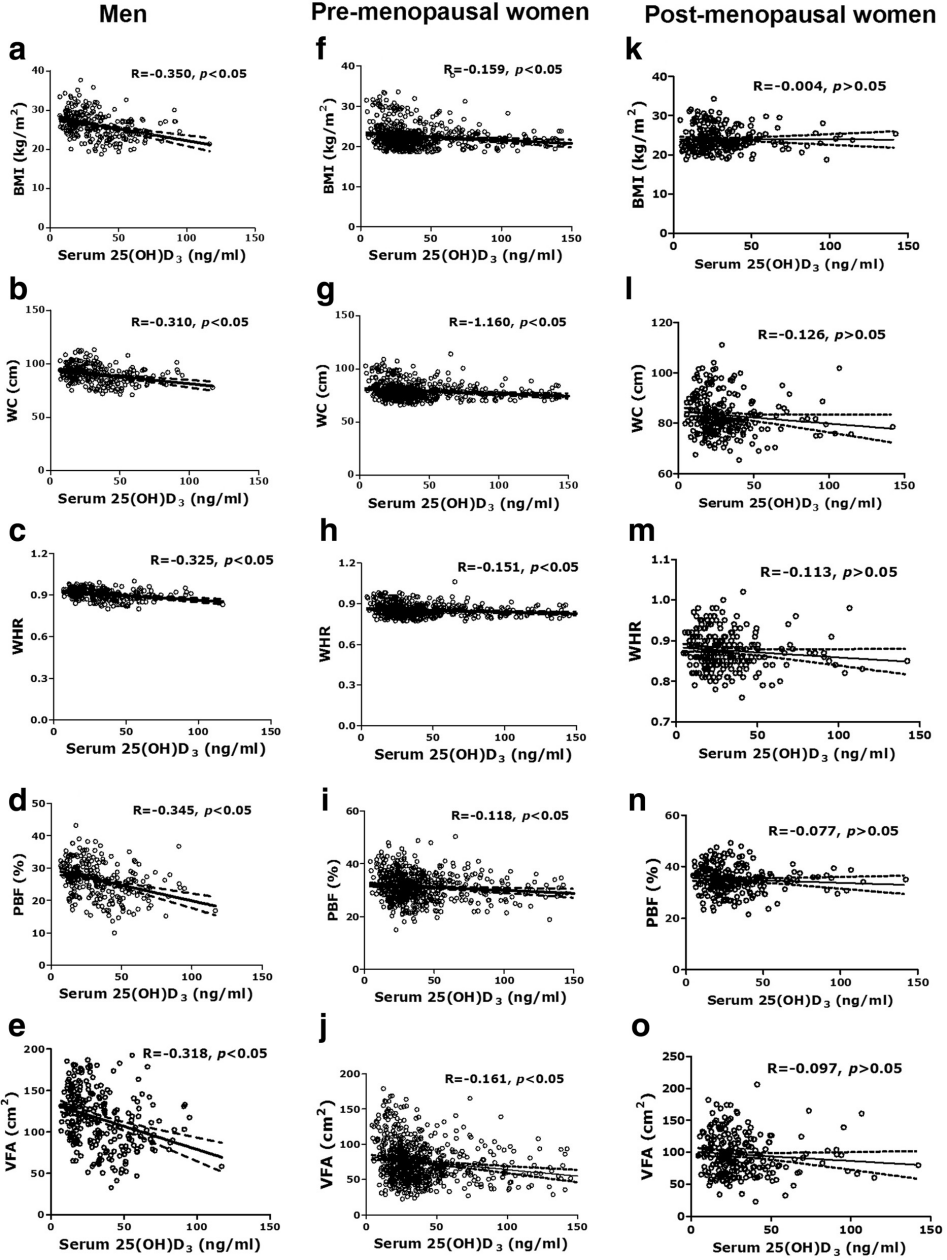
The correlation between serum 25(OH) D_3_ and fatness indices in men (**a**-**e**) and women [pre-menopausal women (**f**-**j**); post-menopausal women **(k-o)**]. Abbreviations: 25(OH)D_3_, 25-hydroxyvitamin D_3_; BMI, body mass index; WC, waist circumference; WHR, waist-to-hip ratio; PBF, percentage body fat; VFA, visceral fat area

Partial correlation analysis showed that serum 25(OH) D_3_ levels were negatively correlated with VFA and other body fatness indices in men (Fig. 2a-e) and pre-menopausal women (Fig. 2f-j) (all *P* < 0.05), not in post-menopausal women (Fig. 2k-o). We also assessed the odds ratios (ORs) and 95 % confidence intervals (CIs) of VFA levels and vitamin D insufficiency and deficiency. After adjusting for age, smoking status, drinking status, exercise status, sun exposure and lipid profiles, as compared to individuals with the lowest VFA, those who had the highest VFA were at 4.9-fold risk of vitamin D insufficiency and deficiency (95 % CI: 1.792–13.365) in men and 1.8-fold risk of vitamin D insufficiency and deficiency (95 % CI: 1.051–3.210) in pre-menopausal women, not in post-menopausal women (Table 4).

**Table 4:** The odd ratios of different fatness indices and vitamin D insufficiency and deficiency in men and women

Gender	Fatness indices	Crude model		Adjusted model^a^	
		OR (95 % CI)	*P*-Value	OR (95 % CI)	*P*-Value
Men	Visceral fat area				
	Q1(<89.95 cm^2^)	1.0		1.0	
	Q2(89.95–115.72 cm^2^)	2.059(0.990–4.281)	0.053	1.320(0.476–3.662)	0.594
	Q3(115.72–140.46 cm^2^)	3.987(1.900–8.364)	0.000	2.424(0.916–6.417)	0.075
	Q4(≥140.46 cm^2^)	7.667(3.495–16.817)	0.000	4.894(1.792–13.365)	0.002
	Continuous	1.025(1.016–1.034)		1.719(1.251–2.361)	
	*P*-value for trend	0.000		0.001	
	Body mass index				
	<28 kg/m^2^	1.0		1.0	
	≥28 kg/m^2^	3.930(2.162–7.142)	0.000	2.857(1.360–6.001)	0.006
	Waist circumference				
	<90 cm	1.0		1.0	
	≥90 cm	3.007(1.809–4.998)	0.000	2.199(1.130–4.279)	0.020
	Waist-to-hip ratio				
	<0.90	1.0		1.0	
	≥0.90	3.802(2.210–6.539)	0.000	2.052(1.010–4.169)	0.047
Pre-menopausal women	Visceral fat area				
	Q1(<53.15 cm^2^)	1.0		1.0	
	Q2(53.15–66.18 cm^2^)	1.240(0.781–1.968)	0.361	1.097(0.644–1.868)	0.735
	Q3(66.18–84.47 cm^2^)	1.257(0.803–1.068)	0.317	1.215(0.717–2.059)	0.469
	Q4(≥84.47 cm^2^)	2.338(1.478–3.697)	0.000	1.837(1.051–3.210)	0.033
	Continuous	1.013(1.008–1.018)		1.265(1.037–1.543)	
	*P*-value for trend	0.000		0.030	
	Body mass index				
	<28 kg/m^2^	1.0		1.0	
	≥28 kg/m^2^	3.921(1.952–7.875)	0.000	3.052(1.430–6.514)	0.004
	Waist circumference				
	<80 cm	1.0		1.0	
	≥80 cm	1.809(1.297–2.523)	0.000	1.647(1.121–2.419)	0.011
	Waist-to-hip ratio				
	<0.85	1.0		1.0	
	≥0.85	1.487 (1.081–2.046)	0.015	1.281(0.878–1.871)	0.199
Post-menopausal women	Visceral fat area				
	Q1(<71.55 cm^2^)	1.0		1.0	
	Q2(71.55–91.97 cm^2^)	0.813(0.392–1.686)	0.577	0.912 (0.359–2.319)	0.847
	Q3(91.97–115.73 cm^2^)	1.153(0.550–2.418)	0.706	1.254(0.506–3.106)	0.625
	Q4(≥115.73 cm^2^)	1.800(0.829–3.909)	0.137	2.326(0.903–5.991)	0.080
	Continuous	1.010(1.001–1.019)		1.012(1.002–1.022)	
	*P*-value for trend	0.028		0.024	
	Body mass index				
	<28 kg/m^2^	1.0		1.0	
	≥28 kg/m^2^	1.577(0.691–3.598)	0.279	1.379(0.548–3.470)	0.494
	Waist circumference				
	<80 cm	1.0		1.0	
	≥80 cm	1.391(0.814–2.377)	0.228	1.522(0.780–2.969)	0.218
	Waist-to-hip ratio				
	<0.85	1.0		1.0	
	≥0.85	1.413(0.776–2.573)	0.258	1.540(0.732–3.241)	0.255

^a^Adjusted for age, smoking status, drinking status, exercise status, sunshine time and lipid profiles

## Discussion

In this cross-sectional study, we confirmed that visceral fat obesity was an important risk factor of vitamin D insufficiency and deficiency among Chinese men and pre-menopausal women, and discovered the increase of visceral adiposity was positively associated with vitamin D insufficiency or deficiency risk with clear dose–response relationship.

There is evidence that vitamin D metabolism, storage, and action both influence and are influenced by adiposity. Observational studies have reported that an increased risk of vitamin D deficiency in those people with obesity [24]. Active vitamin D (1, 25-dihydroxyvitamin D) may influence the mobilisation of free fatty acids from the adipose tissue [25]. In vitro experiments in rats have also shown that large doses of vitamin D_2_ lead to increases in energy expenditure due to uncoupling of oxidative phosphorylation in adipose tissues [26]. Consistent with the experimental studies, our result has indicated that serum 25(OH) D_3_ levels decreased significantly in subjects with visceral obesity and has shown that this decrease was closely correlated with fat distribution [27, 28].

Although the mechanism for the association between obesity and vitamin D insufficiency is not well understood, researches suggested that individuals with obesity were prone to vitamin D insufficiency [29–31], possibly as the result of increased sequestration of vitamin D in the increased amounts of visceral adipose tissue in individuals with obesity [30]. This may lead to less vitamin D released into the blood, and consequently these individuals may have lower serum 25(OH) D_3_ levels. Such individuals may therefore need to increase their vitamin D intake to attain serum 25(OH) D_3_ levels comparable with individuals with normal BMI [29]. Evidence also suggests that vitamin D insufficiency may stimulate lipogenesis as the result of increased calcium influx into the adipocytes mediated by increased synthesis of parathyroid hormones [32]. This would suggest that vitamin D insufficiency could cause obesity. Minimal outdoor activity and maximum skin covering by clothing among the population with obesity also limits photochemical subcutaneous synthesis of vitamin D [31]. Although we have found an association between VFA and vitamin D insufficiency and deficiency, we used cross-sectional data, and thus, were precluded from establishing a temporal relationship between VFA and vitamin D insufficiency and deficiency. However, clinical trials have failed to conclusively demonstrate the benefits of vitamin D supplementation. Randomized controlled trials testing the effect of vitamin D supplementation on weight loss in individuals with obesity or overweight have provided inconsistent findings [33–35]. Dilution related to the greater volume of distribution has been recently proposed as the most likely explanation for the lower 25(OH) D_3_ concentrations in individuals with obesity [36]. In that study, no evidence was found for reduced bioavailability through increased sequestration of vitamin D in the adipose tissue, which had previously been suggested to contribute to the low 25(OH) D_3_ concentrations in obesity. Therefore, these results suggested that although increased in vitamin D status were not likely to help with weight regulation, increased risk of vitamin D deficiency could contribute to the adverse health effects associated with obesity. Recent study has showed that higher BMI led to lower vitamin D status, providing evidence for the role of obesity as a causal risk factor for the development of vitamin D deficiency on the basis of a bi-directional genetic approach [37].

Next to common adiposity measures, we also discovered that VFA was inversely associated with serum 25(OH) D_3_ concentrations in Chinese men and pre-menopausal women, which was similar to the recent study in Northeast Germany and Denmark [38] and China [16]. Differed from Hao’s study among Chinese population, our study population included men and women, and included the participants who were diabetes. Especially, we have observed that the men had the higher risk for vitamin D insufficiency and deficiency. It was because the prevalence of obesity and visceral obesity in men was higher than in women. Obesity is known to be associated with decreased bioavailability of vitamin D, which is sequestered in body fat [30]. Moreover, there was a clear difference in the relationship of VFA and vitamin D insufficiency and deficiency between post- and pre-menopausal women in our study. On one hand, the prevalence of vitamin D insufficiency and deficiency in our study was higher in post-menopausal women. The vitamin D inadequacy is also present in many postmenopausal women in European, North American countries and Eastern Asia [39–42]. On the other hand, there was no difference in the vitamin D insufficiency and deficiency across the quartiles of VFA among post-menopausal women. Thus, we did not observe a relationship between VFA and vitamin D insufficiency and deficiency in post-menopausal women.

Still other studies have implicated the decreased level of serum 25(OH) D_3_ was considered as a risk factor for obesity and its related metabolic disorders including hypertension, high pulse pressure, obesity, hyperlipidemia, diabetes, etc., which increases the chance of cardiovascular disease [43–48]. Currently, researches on vitamin D and metabolic syndrome or its components, which are known to increase cardiovascular disease, are being conducted all over the world [49–52]. Compared with the non-metabolic syndrome group, serum 25(OH) D_3_ levels were significantly lower in the metabolic syndrome group [52]. Lu et al. [50] have reported that reduced serum 25(OH) D_3_ levels were associated with metabolic syndrome and its components, and in particular, obesity was highly associated with insulin resistance and serum 25(OH) D_3_ when compared to normal weight. Among Korean studies, metabolic syndrome and hypertension were associated with serum 25(OH) D_3_ levels in a study of middle-aged Koreans [51]. In fact, Forouhi et al. reported a significant interaction between 25(OH) D and BMI on the risk for a 10-year increase in HOMA-IR [52]. Moreover, the release of free fatty acids from adipose tissue can induce insulin resistance, whereas 1, 25-hydroxyvitamin D has been shown to counteract the free fatty acid-induced insulin resistance [53]. The stronger association of vitamin D with insulin resistance among the participants with visceral fat obesity suggests that adequate vitamin D status is more important for the prevention of insulin resistance and metabolic syndrome in these individuals. In addition, a larger VFA was strongly related to a higher prevalence of impaired fasting glucose levels [54], diabetes [54], insulin resistance [54], hypertension [55], abnormality of lipid metabolism [56], and metabolic risk factors [56, 57]. In our study, the prevalence of hyperglycemia and hypertriglyceridemia increased with the quartiles of VFA in both genders (Appendix Table 1), and VFA was closely related with the indices of metabolic disorders (Appendix Table 2). The individuals with the higher VFA levels have the higher risk of hyperglycemia and hypertriglyceridemia in both genders (Appendix Table 3). In accordance with our finding, one study has demonstrated that the VFA rather than the WC itself was a major determinant of metabolic syndrome in premenopausal Korean women [58], whereas the WC and VFA were similarly useful in identifying metabolic syndrome in elderly Korean women [59]. Our study highlights VFA might also be a simple and sensitive index in routine use for screening vitamin D deficiency and vitamin D related disease among general.

This study has several limitations. First, the cross-sectional study design precluded the ability to determine a causal relationship between VFA and vitamin D insufficiency and deficiency. Second, the DEXA, CT and MRI were not used to measure visceral fat mass for our study because the participants in our study from the routine health check-up. The DEXA, CT and MRI were not the regular item of health check-up since they require expensive and specialized equipment, and exposure to radiation. Third, the intact parathyroid hormone (iPTH) is also expected to be a relevant confounder or even the causal factor of the association between VFA and 25(OH) D_3_, and our study often concentrated exclusively on 25(OH) D_3_ or iPTH without considering the interaction between both. In addition, menopausal status was based on self-reporting. Therefore, further cohort study will be conducted to observe the causal relations between VFA and vitamin D insufficiency and deficiency.

## Conclusion

In conclusion, results of our study revealed that higher VFA increases the risk of vitamin D insufficiency and deficiency in men and pre-menopausal women, but not in post-menopausal women. VFA is a better and convenience surrogate marker for visceral adipose measurement and could be used in identifying the risk of vitamin D insufficiency and deficiency in routine health examination.

## Appendix Table 1

**Table 5:** Prevalence of components of metabolic syndrome according to quartiles of visceral fat area

	Men				
	Quartiles of visceral fat area (cm^2^)				*P*-value^a^
	Q1(<89.95)	Q2(89.95-115.72)	Q3(115.72-140.46)	Q4(≥140.46)	
*n*	64	65	64	64	
Hyperglycemia	5(7.81)	20(30.77)^#^	17(26.56)^#^	19(29.69)^#^	0.006
Hypertriglyceridemia	12(18.75)	21(32.31)	28(43.75)^#^	31(48.44)^#^	0.002
Low HDL-C	14(21.88)	19(29.23)	23(35.94)	19(29.69)	^b^NS
High blood pressure	12(18.75)	16(24.62)	16(25.00)	19(29.68)	^b^NS
	Pre-menopausal women				
	Quartiles of visceral fat area (cm^2^)				*P*-value^a^
	Q1(<53.15)	Q2(53.15–66.18)	Q3(66.18–84.47)	Q4(≥84.47)	
*n*	153	144	163	153	
Hyperglycemia	2(1.31)	8(5.56)	16(9.82)^#^	38(24.84)^#^^&^^$^	0.000
Hypertriglyceridemia	2(1.31)	8(5.56)	19(11.66)^#^	29(18.95)^#^^&^	0.000
Low HDL-C	41(26.80)	54(37.50)	76(46.63)^#^	93(60.78)^#^^&^^$^	0.000
High blood pressure	2(1.31)	4(2.77)	11(6.75)^#^	21 (13.73)^#^^&^^$^	0.000
	Post-menopausal women				
	Quartiles of visceral fat area (cm^2^)				*P*-value^a^
	Q1(<71.55)	Q2(71.55–91.97)	Q3(91.97–115.73)	Q4(≥115.73)	
*n*	59	59	59	58	
Hyperglycemia	12(20.34)	22(37.29)	26(44.07)^#^	22(37.93)^#^	0.045
Hypertriglyceridemia	7(11.86)	10(16.95)	20(33.90)^#^	24(41.38)^#^^&^	0.001
Low HDL–C	13(22.03)	20(33.90)	28(47.46)^#^	26(44.83)^#^	0.017
High blood pressure	12(18.64)	17(28.81)	15(25.42)	19(32.76)	^b^NS

The metabolic risk factors were defined as follows: (1) high blood pressure, with systolic blood pressure of at least 130 mmHg or diastolic blood pressure of at least 85 mmHg or taking medication for previously diagnosed hypertension; (2) hypertriglyceridemia, with TG levels of at least 150 mg/dL (1.69 mmol/L); (3) low HDL-C, with HDL cholesterol level less than 40 mg/dL (1.03 mmol/L) for men and less than 50 mg/dL (1.29 mmol/L) for women; (4) hyperglycemia, with fasting plasma glucose of at least 110 mg/dL (6.1 mmol/L) or taking medication for previously diagnosed type 2 diabetes

Values are number (percentages)

^a^Comparison among four groups

^b^NS = no significant at *p* value > .05

^#^Compared with Q1, *P* < 0.05

^&^Compared with Q2, *P* < 0.05

^$^Compared with Q3, *P* < 0.05

## Appendix Table 2

**Table 6:** Correlation coefficients of the relationships between visceral fat area and various parameters of metabolic risk factors

Indices	Men		Pre-menopausal women		Post-menopausal women	
	r	*P*-value	r	*P*-value	r	*P*-value
Body mass index	0.763	0.000	0.719	0.000	0.818	0.000
Waist circumference	0.818	0.000	0.949	0.000	0.879	0.000
Waist-to- hip ratio	0.899	0.000	0.893	0.000	0.930	0.000
Systolic blood pressure	0.112	0.152	0.283	0.000	0.221	0.007
Diastolic blood pressure	0.162	0.038	0.296	0.000	0.236	0.004
Total cholesterol	0.211	0.007	0.218	0.000	−0.013	0.842
Triglycerides	0.140	0.025	0.382	0.000	0.262	0.000
High-density lipoprotein cholesterol	−0.198	0.011	−0.240	0.000	−0.254	0.000
Low-density lipoprotein cholesterol	0.326	0.000	0.297	0.000	0.004	0.957
Fasting plasma glucose	0.128	0.040	0.259	0.000	0.105	0.107

## Appendix Table 3

**Table 7:** Multivariate analysis of the ORs and 95 % CIs between visceral fat area and components of metabolic syndrome

	Quartiles of visceral fat area			
	Q1	Q2	Q3	Q4
Men				
Hyperglycemia	1.0	6.735(1.642–27.768)	3.996(0.940–16.979)	4.474(1.066–18.780)
Hypertriglyceridemia	1.0	1.815(0.713–4.621)	3.229(1.268–8.222)	3.418(1.358–8.602)
Low HDL-C	1.0	1.935(0.715–5.237)	2.383(0.871–6.519)	1.935(0.703–5.327)
High blood pressure	1.0	4.205(0.808–21.833)	3.707(0.715–19.203)	4.796(0.916–25.104)
Pre-menopausal women				
Hyperglycemia	1.0	3.179(0.634–15.944)	3.920(0.824–18.659)	10.578(2.337–47.874)
Hypertriglyceridemia	1.0	4.441(0.927–21.277)	9.962(2.280–43.535)	17.657(4.132–75.460)
Low HDL-C	1.0	1.481(0.852–2.576)	2.103(1.219–3.628)	3.846(2.146–6.892)
High blood pressure	1.0	1.539(0.131–18.020)	1.415(0.116–17.228)	5.241(0.556–49.437)
Post-menopausal women				
Hyperglycemia	1.0	3.396(1.104–10.447)	4.205(1.408–12.560)	3.867(1.304–11.466)
Hypertriglyceridemia	1.0	2.456(0.626–9.636)	4.602(1.265–16.735)	8.402(2.402–29.391)
Low HDL-C	1.0	2.293(0.860–6.110)	1.944(0.749–5.047)	1.751(0.680–4.509)
High blood pressure	1.0	1.502(0.326–6.919)	1.489(0.354–6.257)	1.607(0.408–6.330)

Odds ratios and 95 % CIs adjusted for age, smoking status, drinking status, exercise status, sunshine time. Men (Q1: <89.95 cm^2^, Q2: 89.95–115.72 cm^2^, Q3: 115.72–140.46 cm^2^, Q4: ≥140.46 cm^2^); Pre-menopausal women (Q1: <53.15 cm^2^, Q2: 53.15–66.18 cm^2^, Q3: 66.18–84.47 cm^2^, Q4: ≥ 84.47 cm^2^); Post-menopausal women (Q1: <71.55 cm^2^, Q2: 71.55–91.97 cm^2^, Q3: 91.97–115.73 cm^2^, Q4: ≥115.73 cm^2^)

## Abbreviations

VFO: Visceral fat obesity
VFA: Visceral fat area
25(OH) D_3_: 25-hydroxyvitamin D_3_
BMI: Body mass index
WC: Waist circumference
WHR: Waist-to-hip ratio
FM: Fat mass
FFM: Free fat mass
PBF: Percentage body fat
DEXA: Dual-energy x-ray absorptiometry
MRI: Magnetic resonance imaging
CT: Computed tomography
BIA: Bioelectrical impedance analysis
FPG: Fasting plasma glucose
TC: Total cholesterol
TG: Triglycerides
LDL-c: Low-density lipoprotein cholesterol
HDL-c: High-density lipoprotein cholesterol
HPLC: High-performance liquid chromatography
ANOVA: One-way analysis of variance
